# Comparison of the Use of Arterial Doppler Waveform Classifications in Clinical Routine to Describe Lower Limb Flow

**DOI:** 10.3390/jcm10030464

**Published:** 2021-01-26

**Authors:** Antoine Guilcher, Damien Lanéelle, Clément Hoffmann, Jérôme Guillaumat, Joel Constans, Luc Bressollette, Claire Le Hello, Christian Boissier, Alessandra Bura-Rivière, Vincent Jaquinandi, Loukman Omarjee, Philippe Lacroix, Gilles Pernod, Fabrice Abbadie, Marie Antoinette Sevestre, Carine Boulon, Guillaume Mahé

**Affiliations:** 1 Clinical Investigation Center, Univ Rennes, INSERM CIC 1414, CHU Rennes, F-35033 Rennes, France; antoine.guilcher@chu-rennes.fr (A.G.); vincent.jaquinandi@chu-rennes.fr (V.J.); loukman.omarjee@chu-rennes.fr (L.O.); 2 Vascular Medicine Unit, CHU Caen-Normandie, F-14000 Caen, France; laneelle-d@chu-caen.fr (D.L.); jerome.guillaumat@chu-caen.fr (J.G.); 3 Vascular Medicine Unit, CHU Brest, F-29200 Brest, France; clement.hoffmann@chu-brest.fr (C.H.); luc.bressollette@chu-brest.fr (L.B.); 4 Vascular Medicine Unit, CHU Bordeaux, F-33076 Bordeaux, France; joel.constans@chu-bordeaux.fr (J.C.); carine.boulon@chu-bordeaux.fr (C.B.); 5 Vascular Medicine Department, CHU Nord Saint-Etienne, Campus Health and Innovations, Jean Monnet University, F-42055 Saint-Etienne, France; claire.lehello@chu-st-etienne.fr (C.L.H.); christian.boissier@chu-st-etienne.fr (C.B.); 6 Vascular Medicine Unit, CHU Toulouse, F-31000 Toulouse, France; bura-riviere.a@chu-toulouse.fr; 7 Vascular Medicine Unit, CHU Limoges, F-87000 Limoges, France; philippe.lacroix@unilim.fr; 8 Vascular Medicine Unit, CHU Grenoble, F-38000 Grenoble, France; GPernod@chu-grenoble.fr; 9 Vascular Medicine Unit, CH Vichy, F-03200 Vichy, France; fabrice.abbadie@ch-vichy.fr; 10 Vascular Medicine Unit, CHU Amiens, F-80054 Amiens, France; sevestre.marie-antoinette@chu-amiens.fr

**Keywords:** peripheral artery disease, Doppler, methods, vascular medicine

## Abstract

Background: Characterisation of arterial Doppler waveforms is a persistent problem and a source of confusion in clinical practice. Classifications have been proposed to address the problem but their efficacy in clinical practice is unknown. The aim of the present study was to compare the efficacy of the categorisation rate of Descotes and Cathignol, Spronk et al. and the simplified Saint-Bonnet classifications. Methods: This is a multicentre prospective study where 130 patients attending a vascular arterial ultrasound were enrolled and Doppler waveform acquisition was performed at the common femoral, the popliteal, and the distal arteries at both sides. Experienced vascular specialists categorized these waveforms according to the three classifications. Results: of 1033 Doppler waveforms, 793 (76.8%), 943 (91.3%) and 1014 (98.2%) waveforms could be categorized using Descotes and Cathignol, Spronk et al. and the simplified Saint-Bonnet classifications, respectively. Differences in categorisation between classifications were significant (Chi squared test, *p* < 0.0001). Of 19 waveforms uncategorized using the simplified Saint-Bonnet classification, 58% and 84% were not categorized using the Spronk et al. and Descotes and Cathignol classifications, respectively. Conclusions: The results of the present study suggest that the simplified Saint-Bonnet classification provides a superior categorisation rate when compared with Spronk et al. and Descotes and Cathignol classifications.

## 1. Introduction

Lower extremity Peripheral artery disease (PAD) prevalence increases with age, affecting more than one in ten people aged 70 years [1]. Moreover, PAD prevalence is very likely underestimated as studies suggest that up to half of the patients suffering from PAD are asymptomatic [2,3,4]. With an aging population, PAD is a significant public health issue and a financial burden on the wider society [5,6,7,8].

The current gold standard for PAD diagnosis is the ankle brachial pressure index (ABPI) [9,10], which is the ratio of the higher of the two systolic blood pressure (SBP) readings measured at the posterior tibial and the pedis dorsalis arteries, to the highest brachial SDP taken at both arms, where an ABPI value of equal or less than 0.90 denotes PAD. However, ABPI has limitations, especially in patients with arterial stiffness such as those suffering from diabetes mellitus and chronic kidney disease. In these patients, peripheral artery walls are stiffened by calcification leading to an overestimation of ABPI [11,12]. 

The toe-brachial pressure index (TBPI) [9,10], which is the ratio of posterior tibial or pedis dorsal artery SBP to brachial SBP, is recommended as an alternative to ABPI in populations with stiffened arteries. However, evidence for the reliability of the method and its added value compared to ABPI is sparse in the literature [13,14].

Doppler waveform analysis is another tool used to diagnose PAD when one cannot rely on ABPI as suggested by recent American Heart Association (AHA), European Society of Cardiology/ European Society for Vascular Surgery (ESC/ESVS) guidelines [9,15]. There is extensive literature showing the relationship between Doppler waveform contours and PAD [16,17,18,19]. However, these waveform contours can be interpreted differently by different physicians and are often a source of confusion and misunderstanding [20,21,22], potentially leading to suboptimal care. Therefore, adding a level of subjectivity in the diagnosis of PAD. For harmonization purposes, classifications have been proposed to define and categorize Doppler waveforms and relate these to different stages of arterial wall damage. In 1975, Descotes and Cathignol were the first to propose a classification [23]. Spronk et al. proposed another classification in 2005 [24]. More recently, in 2017, the French college of vascular medicine teachers (CEMV) suggested the Saint-Bonnet classification and its simplified version [25].

However, little is known about the ability of classifications to categorize arterial Doppler waveforms. The categorization rate of the classification, or efficacy of a classification, depends on how many waveforms can be identified as belonging to a category described within a classification. This is a key parameter of a classification performance as it is a reflection of the relevance of a classification. The aim of the present study was to compare the efficacy of the three aforementioned classifications to categorize Doppler waveforms.

## 2. Experimental Section

### 2.1. Study Design and Population

In this prospective, multicentre study 130 patients were recruited at seven hospitals across France. All patients over 18 years of age and attending a vascular arterial ultrasound were enrolled. Age, gender, blood pressure, ABPI, medical history of cardiovascular disease (history of coronary artery disease, arterial angioplasty and stroke) and cardiovascular risk factors (history of arterial hypertension (AHT), diabetes and dyslipidaemia as well as smoking status) were recorded. Patients were documented as suffering from arterial hypertension if they were prescribed a hypertensive drug, patients were documented as suffering from dyslipidaemia if they were prescribed a lipid lowering treatment.

The Fontaine scale was used to grade the severity of PAD [26]. The grading was done after patient interrogation and clinical examination.

ABPI was measured using the procedure described in the AHA guidelines [27].

**Figure 1:**
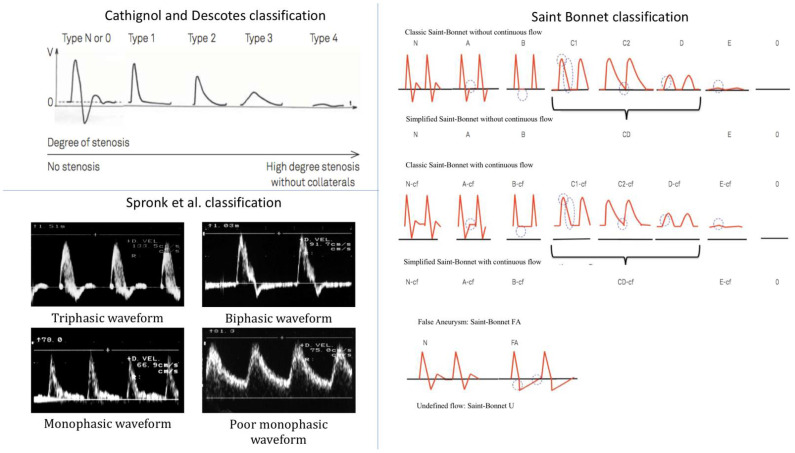
The different arterial Doppler waveform classifications. Panel labelled Cathignol and Descotes classification (adapted from [23]): Type N or 0 is considered as a normal waveform. Panel labelled Spronk et al. classification (adapted from [24]): Triphasic of biphasic types are considered normal. Monophasic and poor monophasic types describe different levels of peripheral artery disease (PAD), from the least to the highest degree. Panel labelled simplified Saint-Bonnet classification: Types N and A are considered normal. Types B to E describes different levels of PAD, from the least to the highest degree.

**Figure 2:**
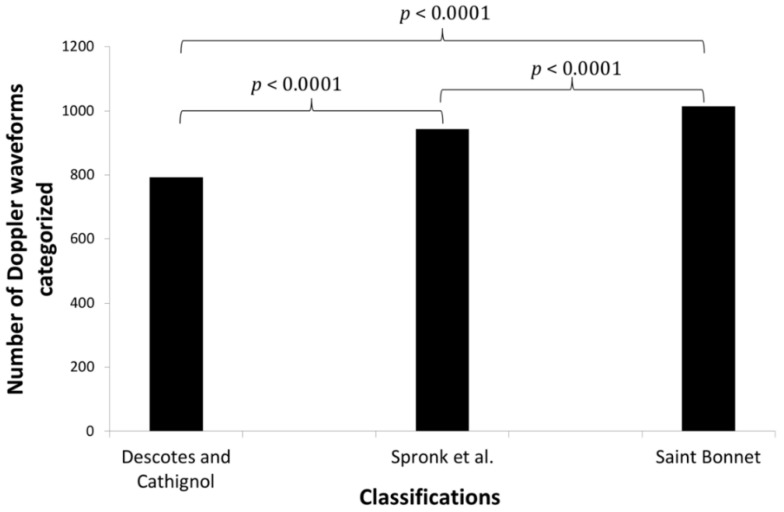
Differences in categorization between the different classifications. Side-by-side comparison of the three classifications categorization rate. *p* values according to Chi squared test.

**Figure 3:**
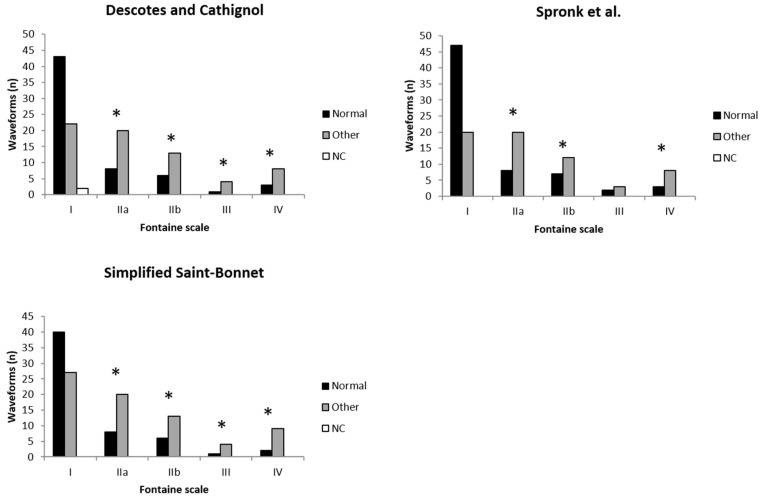
Number of normal and abnormal waveforms according to the Fontaine scale. In this case, waveforms refer to the lower-rated waveforms across both limbs according to each classification. Normal waveforms: Descotes and Cathignol type N, Spronk et al.’s triphasic and biphasic types and simplified Saint-Bonnet types N and A. Other waveforms: categorized but not normal, as defined above. NC: not categorized. * *p* < 0.05 compared with Fontaine grade I.

Doppler waveforms were acquired by 11 vascular medicine physicians with at least three years’ experience in arterial Doppler ultrasonography and who were not familiar with the simplified Saint-Bonnet, Descotes and Cathignol and Spronk et al. classifications, as prior to 2017 the French vascular medicine society did not provide guidelines about the use of arterial Doppler waveform classifications. Arterial Doppler flow waveforms were acquired across all seven centres, at the common femoral, the popliteal (infragenual), the anterior tibial and the pedis dorsalis arteries at both sides. Categorization of the arterial Doppler waveforms was performed shortly after acquisition. Each of the 11 physicians only categorized the waveforms they acquired. The categorization process was done according to Descotes and Cathignol, Spronk et al. and the simplified Saint-Bonnet classifications. Physicians were not accustomed to any classifications up to one month prior to the start of the study. At that time, posters consisting of Figure 1, Figure 2 and Figure 3 were displayed in every consultation rooms for them to familiarise themselves with the classifications.

The efficacy, or categorization rate, of a classification was defined as the number of waveforms categorized, when using a given classification, over the total number of waveforms.

### 2.2. Classifications

#### 2.2.1. Descotes and Cathignol Classification

This classification was first proposed by Descotes and Cathignol in 1975 and distinguishes between five types of Doppler waveforms, from normal (type N) to the most pathological (type 4) (Figure 1) [23].

Type N or 0 describes a triphasic waveform, characteristic of healthy resistive arteries, with a systolic velocity peak followed by a reflux phase, ending with a small diastolic velocity peak. Type 1 describes a monophasic waveform and differs from type N with the disappearance of the reflux and the diastolic velocity peak. Type 2 describes an attenuated type 1 waveform with an enlargement of the systolic peak. Type 3 describes a further attenuated waveform with a further enlargement of the systolic peak. Type 4 describes a waveform where flow velocity is almost zero.

#### 2.2.2. Spronk et al. Classification

Spronk et al. first proposed this classification in 2005 by distinguishing between four types of Doppler arterial flow waveforms according to the number of phases during a heart cycle (Figure 1), a lower number of phases being associated with a more pathological artery [24].

The triphasic type is very similar to Descotes and Cathignol type N. It corresponds to a Doppler waveform made of three phases: a sharp systolic forward rise and fall, an element of reverse flow during diastole, and an element of forward flow during diastole. The biphasic type describes a two-phase Doppler waveform with an extension of the reverse flow phase during the whole diastole and the disappearance of the forward flow phase during diastole, in comparison with the triphasic type. Tri- and biphasic types are considered normal. The sharp monophasic type consists of a sharp systolic rise, a lack of a diastolic reverse flow element with the presence of a continuous forward flow during diastole. The poor (blunted) monophasic type illustrates a loss of sharpness in the systole with a slow fall after the systolic peak that continues until the end of diastole.

#### 2.2.3. The Simplified Saint-Bonnet Classification

This classification was first proposed in 2017 and differs from the previous classifications by the number of waveform types. The simplified Saint-Bonnet classification distinguishes between 13 types of waveforms [25]. It adds a flow description for false aneurysm waveform and a type for undefined waveforms. It also makes the distinction between waveforms with and without continuous flow (–CF) (Figure 1). It goes from type N to E where N stands for normal and type E describes the type of waveforms recorded in very pathological arteries.

Type N describes a triphasic waveform, very much like Descotes and Cathignol type N and Spronk et al.’s triphasic type. Type A describes a biphasic waveform with the disappearance of the forward flow phase in diastole compared with type N. Types N and A are considered normal. Type B describes a sharp monophasic waveform with the disappearance of the backward flow phase in comparison with type A. Type CD describes an attenuation of type B waveforms with a loss of sharpness of the systolic velocity peak defined as a rounder waveform with a longer velocity fall time. Type E corresponds to a much-attenuated Doppler waveform with a flow velocity close to zero. A further five types are described. They share the same characteristics as the five types described above but with a continuous flow component and are thus labelled with the suffix “–CF”. This continuous flow component is encountered in conditions where peripheral vessel resistance is low, for instance, poststenosis or during exercise. In addition to these 10 types, three more types are described. Type 0 illustrates the absence of flow. Type FA describes a typical Doppler flow waveform seen in case of the presence of a false aneurysm (FA), where the area under the curve in the systole equals the area over the curve in the diastole. Finally, type U is for Doppler waveforms that do not meet the classification criteria of the previously described types.

The undefined type (type U) of the simplified Saint-Bonnet classification has no equivalence in the Descotes and Cathignol and Spronk et al. classifications. As this could introduce an obvious bias in favour of the simplified Saint-Bonnet classification, while not practically improving Doppler waveform categorization, it was decided to omit the use of type U. Although there is no category illustrating the absence of flow in the Descotes and Cathignol and Spronk et al. classifications, its existence is implicit and was made explicit.

### 2.3. Statistics

Statistical analysis was performed using MedCalc (version 18.5, 64 bits, MedCalc Software, Ostend, Belgium). Results are expressed in mean ± standard deviation (SD) in case of normal distribution (Shapiro–Wilk test). Differences in categorization rate between the three classifications were compared using the Chi squared test, where each classification was compared separately to the other two classifications with the number of categorized and non-categorized waveforms used as inputs. A *p*-value < 0.05 was considered significant.

## 3. Results

One hundred and thirty patients were recruited, among which 62% (*n* = 81) were male. All continuous variables were normally distributed (*p* < 0.006). The age of patients ranged from 32 to 92 years. The mean (±SD) ABPI was 1.01 ± 0.40 on the right lower limb and 0.98 ± 0.39 on the left lower limb. Fifty one percent of the patients (*n* = 66) had a history of cardiovascular disease and 98.5% of the patients (*n* = 128) had at least one cardiovascular risk factor (Table 1).

Of a possible 1040 waveforms per patient, a total of 1033 Doppler waveforms were acquired, meaning that seven (0.07%) Doppler waveforms could not be recorded, four of which (0.04%) because a patient had his left limb amputated and three (0.03%) because of nonoptimal exam conditions. Out of 1033 Doppler waveforms, 793 (76.8%), 943 (91.3%) and 1014 (98.2%) waveforms could be categorized using Descotes and Cathignol, Spronk et al. and the simplified Saint-Bonnet classifications, respectively. When comparing the simplified Saint-Bonnet classification to each of the other two classifications the difference in the number of waveforms categorised was significant, with a *p*-value < 0.0001 in both comparisons (Figure 2).

Aside from type E of the simplified Saint-Bonnet classification, there was at least one waveform in each category of all three classifications (Table 2).

Out of the 19 waveforms that could not be categorised using the simplified Saint-Bonnet classification, 58% (*n* = 11) and 84% (*n* = 16) could not be categorised using the Spronk et al. and Descotes and Cathignol classifications, respectively. Similarly, out of the 90 waveforms that were not categorised using the Spronk et al. classification, 12% (*n* = 11) and 71% (*n* = 64) were not categorised according to the simplified Saint-Bonnet and Descotes and Cathignol classifications, respectively, and, out of the 240 waveforms that were not categorised using Descotes and Cathignol’s classification, 7% (*n* = 16) and 27% (*n* = 64) were not categorised using the simplified Saint-Bonnet and Spronk et al. classifications, respectively (Table 3).

Among the 240 waveforms not categorized according to Descotes and Cathignol, 64% (*n* = 154) were categorized as “biphasic” according to Spronk et al.’s classification and 70% (*n* = 168) were categorized as type A according to the simplified Saint-Bonnet classification. Similarly, out of the 90 waveforms not categorized according to Spronk et al.’s classification, 20% (*n* = 18) were categorized as type 2 according to the Descotes and Cathignol classification, 30% (*n* = 27) as type N-CF, and 27% (*n* = 24) as CD according to the simplified Saint-Bonnet classification. When looking at the 19 waveforms not categorized by the simplified Saint-Bonnet classification, four of them (21%) were categorized as biphasic according to Spronk et al. classification. 

When comparing classification categorization of the limiting flow waveform (i.e., the lowest rated waveform across both limbs) against the Fontaine clinical scale, the majority of waveforms (64%, 70% and 60% for the Descotes and Cathignol, Spronk et al. and the simplified Saint Bonnet classifications, respectively) are considered normal in asymptomatic patients. Conversely, up to only 32%, 40% and 32% of waveforms are considered abnormal according to the Descotes and Cathignol, Spronk et al. and the simplified Saint-Bonnet classifications, respectively. This waveform distribution is inverted when patients are symptomatic according to the Fontaine scale, with a majority of waveforms being categorised as abnormal (*p* < 0.05, apart for grade III patients with the Spronk et al. classification) (Figure 3).

## 4. Discussion

The results from the present study suggest that of the three classifications studied the simplified Saint-Bonnet classification provides the best Doppler waveform categorization rate. 

The main difference with the simplified Saint-Bonnet classification compared to the other two classifications is the number of categories it includes. When Doppler waveforms were not categorised according to the simplified Saint-Bonnet classification, most of these waveforms (58% and 84%) were not categorised, according to the Spronk et al. and Descotes and Cathignol classifications, respectively (Table 3). Reciprocally, 88% and 98% of the Doppler waveforms that were not categorized using the Spronk et al. and Descotes and Cathignol classifications, respectively, were categorized using the simplified Saint-Bonnet classification. This illustrates the advantage of a higher number of categories, describing a wider range of waveforms profiles encountered in clinical practice. However, a higher number of categories does not necessarily relate to a better classification rate. This is highlighted when comparing the categorisation rates of the Descotes and Cathignol and Spronk et al. classifications. Although the Descotes and Cathignol classification has a higher number of categories, its rate of waveform categorisation is significantly lower. 

Another difference between the simplified Saint-Bonnet, Descotes and Cathignol and Spronk et al. classifications is the waveform description used to define categories. Biphasic or type A Doppler waveforms, according to Spronk et al. and the simplified Saint-Bonnet classifications, are very similar in their descriptions. The categorization results detailed in Table 2 show that biphasic or type A Doppler waveforms had a prevalence of around 25% in the present study. However, Descotes and Cathignol classification does not include a category describing this type of Doppler waveforms. Furthermore, unlike the simplified Saint-Bonnet classification with type “–CF” and Spronk et al. classification with the “poor monophasic type” and arguably with the ambiguous “sharp monophasic” type, Descotes and Cathignol classification does not include Doppler waveforms with a continuous forward flow. However, such a type of waveform had a prevalence of around 20% in the present study (Table 2). In contrast, the Descotes and Cathignol classification, with types 2, 3 and 4, focuses on the description of waveforms seen in severe PAD, with a low prevalence in the present study. These types of waveforms find an equivalent in the simplified Saint-Bonnet classification with types CD and E, but not in the Spronk et al. classification. 

Therefore, in addition to the number of categories, the definition of the different waveform types and the prevalence of those types in the population studied have an impact on the categorization rate of classifications. 

Several studies have shown the lack of coherence among vascular specialists when describing Doppler waveforms [22,28]. This is a source of confusion and can lead to sub-optimal care. While classifications are a useful tool to harmonise Doppler waveform analysis, the results of the present study suggest interobserver variability may still be an issue. Indeed, while types N from the simplified Saint-Bonnet and Descotes and Cathignol classifications and “triphasic” type from Spronk et al. are very similar in their description, the number of Doppler waveforms categorized as such differs (Table 2). This highlights the ambiguity of classifications. It could originate from the wording of definitions used to categorise waveforms or the iconography chosen to illustrate each category (schematics versus real images). Part of that ambiguity might be dealt with by proper training in using classifications. Nonetheless, this discrepancy particularly stresses the need to evaluate inter- and intraoperator variability of classifications, of which no data is found in literature.

Despite not being the main objective of the study, all three classification ratings seem to correlate with clinical symptoms (Figure 3). This result was expected as several studies have shown a relationship between arterial Doppler waveform contour and PAD [16,17,18,19]. Nonetheless, it suggests that the classes descriptions of the three classifications assessed in the present study are clinically relevant. In order to further explore the relationship between classifications’ categories and the degree of severity of PAD, studies assessing the degree of stenosis and the immediate downstream flow contour are needed. If conclusive, the results could reinforce the use of waveform classifications in assessing the severity of PAD.

Another interesting result is the lack of significant difference between Fontaine grades I and III when comparing the proportion of normal and abnormal waveforms categorized according to the Spronk et al. classification (Figure 3). This result could be the consequence of the lower number of categories comprised in the Spronk et al. classification. Possibly, an intermediate category is lacking. If present, it would allow for the differentiation between normal and abnormal waveforms in patient suffering from grade III PAD.

The results of the present study (Figure 3) also highlight that a significant narrowing, leading to a lower rated Doppler waveform, can be present without any symptoms. Indeed, around half of the asymptomatic patients had a record of a deteriorated Doppler flow waveform (Figure 3). This number is in line with previous studies [2,3,4].

The present study has several limitations. The categorisation order was not assessed. It is thus impossible to evaluate the impact of the simplified Saint-Bonnet classification appearing last in the categorization form. In addition, operator preference towards one classification, which could introduce a bias in favour of the favoured classification, was not assessed. The time taken to categorize waveforms according to the different classification was not assessed either. However, no physician complained about any delay added to their consultation when using either classification as they found the categorization process seamless. Furthermore, as the present study was conducted in a real life set up, inter- and intraobserver variability was not assessed and there was no categorisation cross-examination. Thus, the accuracy of categorization and especially the possibility of overcategorisation (i.e. categorising a waveform that does not fit the definition criteria leading to an overestimation of classification performance) cannot be excluded. Moreover, as underlined in Table 2, prevalence of the different Doppler flow waveform categories varied with a higher proportion of “normal” Doppler waveforms. This is due to the nature of recruitment where no prescreening of patients of the vascular clinic took place, in order to assess the classifications in real practice conditions. Assuming normal waveforms are easier to recognize, their high prevalence in the current study could be possibly be the source of a bias in favour of higher classification rates. Also, as discussed previously, the categorization rate of a classification is very dependent of the categories definition and the prevalence of those categories. In the present study, the prevalence of normal waveform is very likely to create a bias against the Descotes and Cathignol classification compared with the other two classifications. Finally, the current study main objective was to assess the categorisation performance of three classifications and, although the results show a link between Doppler flow waveform categories and PAD symptoms, the analysis remains limited and further work on the matter is required.

## 5. Conclusions

By assessing the categorization rate of the simplified Saint-Bonnet, Descotes and Cathignol and Spronk et al. classifications, the present study is an important first step towards the assessment of the performance of these three classifications, highlighting the validity of the categories defined within the Spronk et al. and the simplified Saint-Bonnet classification. More precisely, the results of the present study suggest that the simplified Saint-Bonnet classification has an advantage over the Descotes and Cathignol and Spronk et al. classifications for categorization.

## Figures and Tables

**Table 1:** Patient characteristics.

Characteristics	Patients (*n* = 130)
Male (%)	81 (62.3)
Female (%)	49 (37.7)
Age (years)	68 ± 11
BMI (kg/m^2^)	26.97 ± 6.2
ABPI	Right: 1.01 ± 0.4 Left: 0.98 ± 0.39
BP (mmHg)	137± 25/75 ± 12
CAD (%)	33 (25.4)
Angioplasty (%)	38 (29.2)
Stroke (%)	17 (13.1)
AHT (%)	94 (72.3)
Dyslipidaemia (%)	82 (63.1)
Diabetes (%)	44 (33.8)
Smokers (%)	43 (33.1)
Fontaine scale	
Grade I (%)	67 (51.5)
Grade IIa (%)	28 (21.5)
Grade IIb (%)	19 (14.6)
Grade III (%)	5 (3.8)
Grade IV (%)	11 (8.5)

SD: standard deviation; ABPI: ankle brachial pressure index; BP: blood pressure; BMI: body mass index; CAD: coronary artery disease; AHT: arterial hypertension.

**Table 2:** Categorization details according to the Saint-Bonnet, Spronk et al. and Descotes and Cathignol classifications.

Simplified Saint-Bonnet		Spronk et al.		Descotes and Cathignol	
N = 1033		N = 1033		N = 1033	
N	389	Triphasic	407	Type N	508
A	283	Biphasic	277	Type 1	85
B	24	Sharp monophasic	124	Type 2	98
CD	64	Poor monophasic	86	Type 3	49
E	0	No flow	49	Type 4	3
N-CF	52	NC	90	No flow	50
A-CF	10			NC	240
B-CF	27				
CD-CF	115				
E-CF	1				
No flow	49				
NC	19				

Simplified Saint-Bonnet types N and A, Spronk et al. tri- and biphasic types and Descotes and Cathignol type N are considered normal waveforms. NC: not categorized; CF: continuous flow. N, A, B, CD, E are different types of Saint-Bonnet classification refer to the text for detailed information.

**Table 3:** Comparison of categorized and not-categorized Doppler waveforms distribution between classifications.

		Saint-Bonnet		Spronk et al.		Descotes and Cathignol	
		NC	C	NC	C	NC	C
Saint-Bonnet							
NC	19			11 (58%)	8 (42%)	16 (84%)	3 (16%)
Spronk et al.							
NC	90	11 (12%)	79 (88%)			64 (71%)	26 (29%)
Descotes and Cathignol							
NC	240	16 (7%)	234 (93)	64 (27%)	176 (73)		

NC: not categorized; C: categorized.

## Data Availability

The data that support the findings of this study are available from the corresponding author upon reasonable request.
